# A compact wideband Wilkinson Power Divider topology for arbitrary *N*-Way outputs

**DOI:** 10.1371/journal.pone.0352515

**Published:** 2026-06-25

**Authors:** Shrawan K. Patel, Rusan Kumar Barik, Niraj Kumar Dewangan, Shweta Kumari, Mrinal K. Mandal

**Affiliations:** 1 Department of Electronics and Communication Engineering, School of Engineering and Technology, GGV Bilaspur, Chhattisgarh, India; 2 Department of Electronics and Communication Engineering, School of Engineering and Technology, CHRIST University, Bangalore, India; 3 Manipal Institute of Technology, Manipal Academy of Higher Education, Manipal, India; 4 Department of Artificial Intelligence, School of Engineering, Anurag University, Hyderabad, India; 5 Department of Electronics and Electrical Communication Engineering, Indian Institute of Technology, Kharagpur, West Bengal, India; Galgotias College of Engineering and Technology, Greater Noida, INDIA

## Abstract

This work presents a compact Wilkinson power divider (WPD) topology capable of realizing arbitrary 1 × *N* equal power divisions without requiring crossovers or unrealizable high-impedance lines. The design combines both equal and unequal power division stages, enabling flexibility for even and odd numbers of outputs. Power divider units with 1:2 and 1:3 ratios are used as the basis for constructing higher-order N-output WPDs. Miniaturization is achieved through tapered-line impedance transformers, limiting the maximum line impedance to 103 Ω within fabrication constraints. A two-stage 1 × 3 divider is designed, fabricated, and tested at 2.4 GHz on a microstrip platform. The prototype demonstrates an 85% effective bandwidth with amplitude imbalance within ±0.2 dB, and phase imbalance less than ±2.5°. Compared to conventional topologies, the present approach achieves up to 58% size reduction while maintaining wideband performance. This work is highly suitable for *M* × *N* high-performance, efficient, reliable, and scalable beamforming network architecture. This work supports SDG 9 Industry Innovation and Infrastructure by enabling compact, scalable, and high-efficiency microwave components for advanced communication and infrastructure systems.

## 1. Introduction

Power dividers and combiners are fundamental passive microwave components widely employed for signal distribution and combination in applications such as RF beamforming, phased arrays, mixers, and power amplifiers [1]. The conventional Wilkinson power divider (WPD) [2] employs two quarter-wavelength transmission lines at the design frequency f_0_. While simple in structure, this approach leads to large circuit sizes, especially at lower frequencies. A single-stage WPD provides narrowband performance, whereas multistage extensions enhance bandwidth at the expense of increased printed circuit board (PCB) area. The number of stages is further constrained by fabrication limits on high-impedance lines.

To avoid the above problem for multiple outputs, a parallel combination of basic 1:2 WPD can be used for even *N*, whereas odd *N* requires one port to be matched, causing power loss. A hybrid of equal and unequal topologies can yield compact and wideband designs. Several microstrip-based approaches for an arbitrary number of outputs have been explored [3–13], including size reduction techniques using electromagnetic band gaps [3], high–low impedance resonators [4], and lumped components [5]. However, all of them suffer from a narrower bandwidth than conventional WPDs.

Multistage topologies are commonly used for wideband WPDs, but they usually result in large circuit sizes. Recent alternatives employ slot or parallel-strip lines [6] and lumped elements [7–9]. Miniaturized unequal WPDs have also been reported [10–13] using few capacitors for matching and phase compensation [10], artificial and double-sided parallel-strip lines [11], stepped-impedance lines with resonators [12], and asymmetric resonators with coupled stubs and folded structures [13]. While these achieve notable size reduction, they become complex at high division ratios.

**Fig 1:**
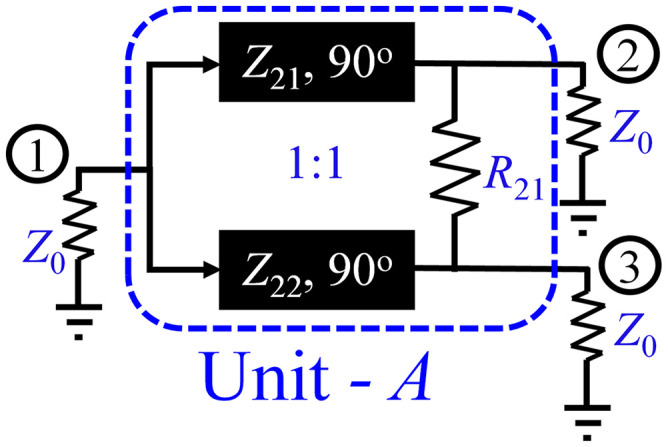
Schematic representation of unit -A (1:2 power division) of proposed WPD.

**Fig 2:**
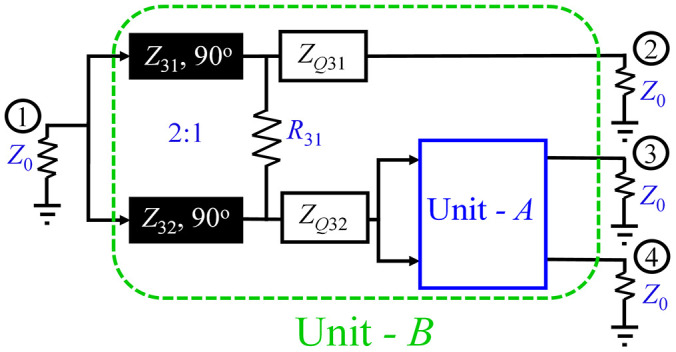
Schematic representation of unit -B (1:3 power division) of proposed WPD.

This article presents a modified WPD topology, which can be used to design and implement any WPD with an arbitrary number of outputs. It avoids crossovers and the limitations due to the implementation of high impedance lines. As shown in Fig 1, a basic 1:2 WPD with *N* = 2 and another 1:3 with *N* = 3, shown in Fig 2, are developed first. Then, any *N* is implementable based on these two basic units with components listed in Table 1.

**Table 1:** Components required in *N*-outputs of present Wilkinson power divider unit for *N* > 3.

No of outputs (*N*)	4	5	6	7	8
**No. of WPD stages ⌈log**_**2**_ **(*N*)⌉**	2	3	3	3	3
**No. of tapered sections 2(*N*-1)**	6	8	10	12	14
**No. of WPD units/resistors (*N*-1)**	3	4	5	6	7

**Fig 3:**
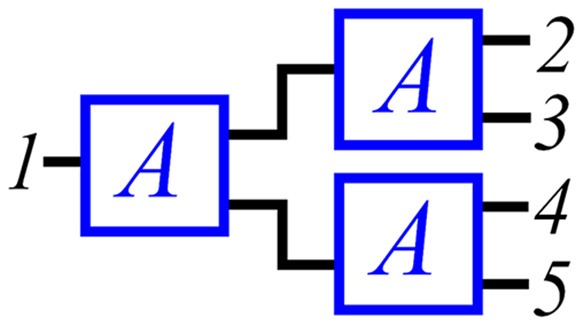
Schematic representation of 1 × 4 WPD configuration.

**Fig 4:**
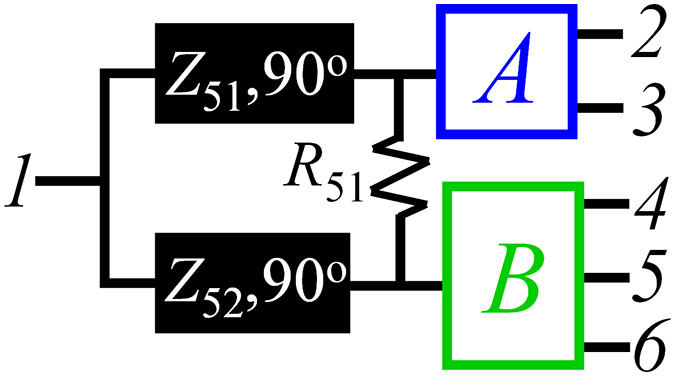
Schematic representation of 1 × 5 WPD configuration.

**Fig 5:**
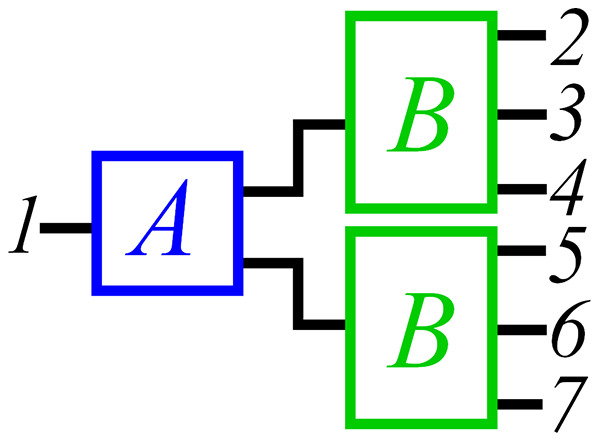
Schematic representation of 1 × 6 WPD configuration.

**Fig 6:**
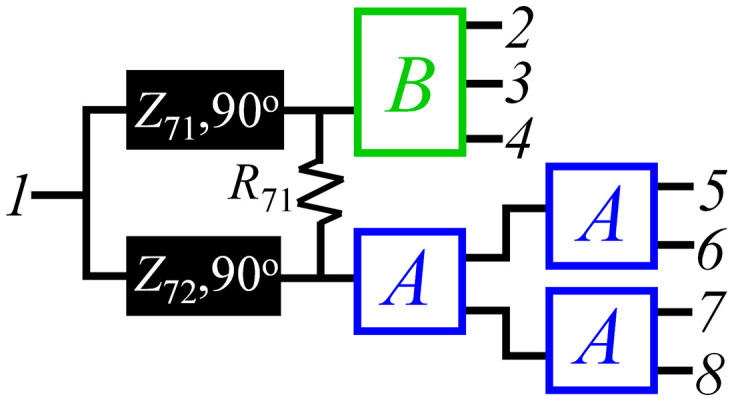
Schematic representation of 1 × 7 WPD configuration.

**Fig 7:**
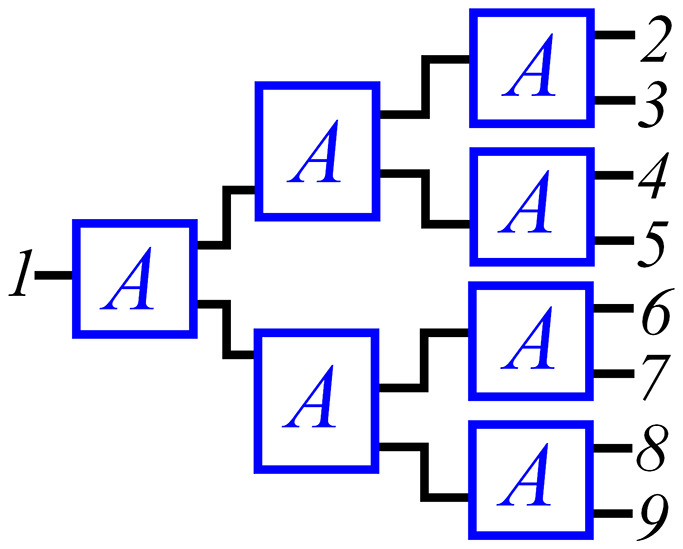
Schematic representation of 1 × 8 WPD configuration.

Various configurations from *N* = 4–8 are shown in Figs 3,4,5, 6 and 7, respectively. A lossless transmission line model is used for all the analysis. Advantages of the present approach compared to others are elaborated. Further, miniaturization is achieved by replacing quarter-wave transformers with tapered lines after each stage for 50 Ω matching. The maximum line impedance is limited to 103 Ω, remaining within fabrication constraints. As *N* increases, the design maintains practical impedance levels through the use of tapered impedance transformers, preserving fabrication feasibility. The taper dimensions, including taper length, width profile, and impedance transition ratio, were optimized through a combination of analytical estimation and full-wave EM simulations.

## 2. Analysis and design of WPD units A and B

The 1:3 power divider (*N* = 3) is further analyzed. While ABCD-parameter-based even- and odd-mode analyses are available in [14,15], the present work employs a simple impedance transformation-based even- and odd-mode analysis, which can be extended to the design of unequal power ratio. The corresponding even- and odd-mode equivalent circuits are shown in Fig 3.

For even mode analysis, a condition to ensure no current flows through resistor *R*_*N*1_ is


$$\begin{array}{c} \overset{e}{\underset{\text{2}}{V}}=\overset{e}{\underset{\text{3}}{V}} \end{array}$$ (1)

If the power division ratio is *P*_2_/*P*_3_ = 1/*k*, to maintain the symmetry, the impedance at node b is


$$\begin{array}{c} \overset{e}{\underset{\text{2}}{Z}}=k\overset{e}{\underset{\text{3}}{Z}} \end{array}$$ (2)

To minimize the impedance transformation ratio for a given power division and thus to maximize the bandwidth, the optimum value of impedances should be chosen, such that their geometric mean is equal to *Z*_0_ [16]. Therefore, even-mode impedances at node b and $b^{\prime }$ are


$$\begin{array}{c} \overset{e}{\underset{\text{2}}{Z}}=Z_{\text{0}}\sqrt{k} \end{array}$$ (3)

and


$$\begin{array}{c} \overset{e}{\underset{\text{3}}{Z}}=Z_{\text{0}}/\sqrt{k} \end{array}$$ (4)

Now, the condition for impedance matching at node b and $b^{\prime }$ is


$$\begin{array}{c} Z_{N1}=kZ_{N2} \end{array}$$ (5)

Since a quarter-wave transformer is used, therefore


$$\begin{array}{c} \overset{e}{\underset{in2}{Z}}=k\overset{e}{\underset{in3}{Z}} \end{array}$$ (6)

The condition for maximum power transfer at node a and $a^{\prime }$ is


$$\begin{array}{c} \frac{\text{1}}{\left(2Z_{\text{0}}\right|\left|2Z_{\text{0}}\right)}=\frac{\text{1}}{\overset{e}{\underset{in2}{Z}}}+\frac{\text{1}}{\overset{e}{\underset{in3}{Z}}} \end{array}$$ (7)

Therefore, from (5) and (6), the input impedances at node a and $a^{\prime }$ are


$$\begin{array}{c} \overset{e}{\underset{in2}{Z}}=Z_{\text{0}}\left(k+1\right) \end{array}$$ (8)

and


$$\begin{array}{c} \overset{e}{\underset{in3}{Z}}=Z_{\text{0}}\left(\frac{k+1}{k}\right) \end{array}$$ (9)

Again, applying the quarter-wave transformer theory in both parallel microstrip lines with characteristics impedances *Z*_*N*1_ and *Z*_*N*2_, the respective expressions are


$$\begin{array}{c} Z_{N1}=Z_{\text{0}}\sqrt{\sqrt{k}\left(k+1\right)} \end{array}$$ (10)

and


$$\begin{array}{c} Z_{N2}=Z_{\text{0}}\sqrt{\left(\frac{\left(k+1\right)}{k\sqrt{k}}\right)} \end{array}$$ (11)

Similarly,


$$\begin{array}{c} Z_{QN1}=Z_{\text{0}}\sqrt{\sqrt{k}} \end{array}$$ (12)

and


$$\begin{array}{c} Z_{QN2}=Z_{\text{0}}\sqrt{\sqrt{\frac{\text{1}}{k}}} \end{array}$$ (13)

Since, in the odd-mode, all current flows through *R*_*N*1_ due to a short-circuit at one end of *Z*_*N*1_ of length 90^o^, the conditions for maximum power transfer at the node b and $b^{\prime }$are


$$\begin{array}{c} R_{\text{2}}=\overset{o}{\underset{\text{2}}{Z}}=\overset{\text{2}}{\underset{QN1}{Z}}/Z_{\text{0}} \end{array}$$ (14)

and


$$\begin{array}{c} R_{\text{3}}=\overset{o}{\underset{\text{3}}{Z}}=\overset{\text{2}}{\underset{QN2}{Z}}/Z_{\text{0}} \end{array}$$ (15)

Hence,


$$\begin{array}{c} R_{\text{2}}=Z_{\text{0}}\sqrt{k} \end{array}$$ (16)

and


$$\begin{array}{c} R_{\text{3}}=Z_{\text{0}}/\sqrt{k} \end{array}$$ (17)

and


$$\begin{array}{c} R_{N1}=Z_{\text{0}}\left(\sqrt{k}+\frac{\text{1}}{\sqrt{k}}\right) \end{array}$$ (18)

Therefore, design steps to implement a 1:3 WPD with *N* = 3, shown in Fig 2, are (i) decide the power division ratio for first stage, i.e., 1:2 (ii) calculate *Z*_31_ and *Z*_32_ using (8) (iii) calculate *Z*_*Q*31_ and *Z*_*Q*32_ using (9) (iv) calculate *R*_31_ using (12) (v) for second stage with 1:1, repeat steps (i) to (iv) to calculate *Z*_21_, *Z*_22_ and *R*_21_ to implement unit-A, shown in Fig 1. (vi) place unit-A at the output port, with double power, of first stage. (vii) design, optimize and fabricate the circuit. For example, calculated parameters of for 1 × 3 WPD unit using present topology are *Z*_21_ = *Z*_22_ = 70.7 Ω, *Z*_31_ = 103 Ω, *Z*_*Q*31_ = 59.45 Ω, *Z*_*Q*32_ = 42.04 Ω, *Z*_32_ = 51.5 Ω, *R*_31_ = 106.05 Ω, and *R*_21_ = 100 Ω.

To miniaturize WPD units, in the present topology, QWTs with impedances *Z*_*Q*31_ and *Z*_*Q*32_ are realized using small tapered microstrip lines to match *Z*_31_ and *Z*_32_ with *Z*_0_, without influencing other performance aspects. With the longitudinal electrical length reduced by 80°, the overall size of the modified 1 × 3 WPD becomes about 70% of a conventional WPD with QWTs. Furthermore, for all configurations having an even number of outputs, the first-stage power division ratio remains 1:1, and a single unit A is sufficient to design a WPD with binary outputs. Conversely, for configurations with an odd number of outputs, the first-stage power division ratio becomes [(*N* + 1)/2]: [(*N*-1)/2].

## 3. Comparison with Conventional *N* – way WPDs

**Fig 8:**
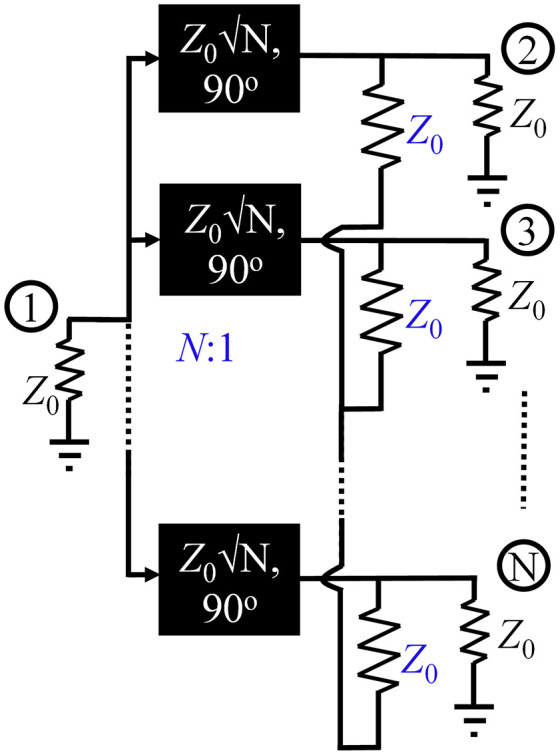
case A: conventional single stage 1 × *N* equal power WPD topology.

**Fig 9:**
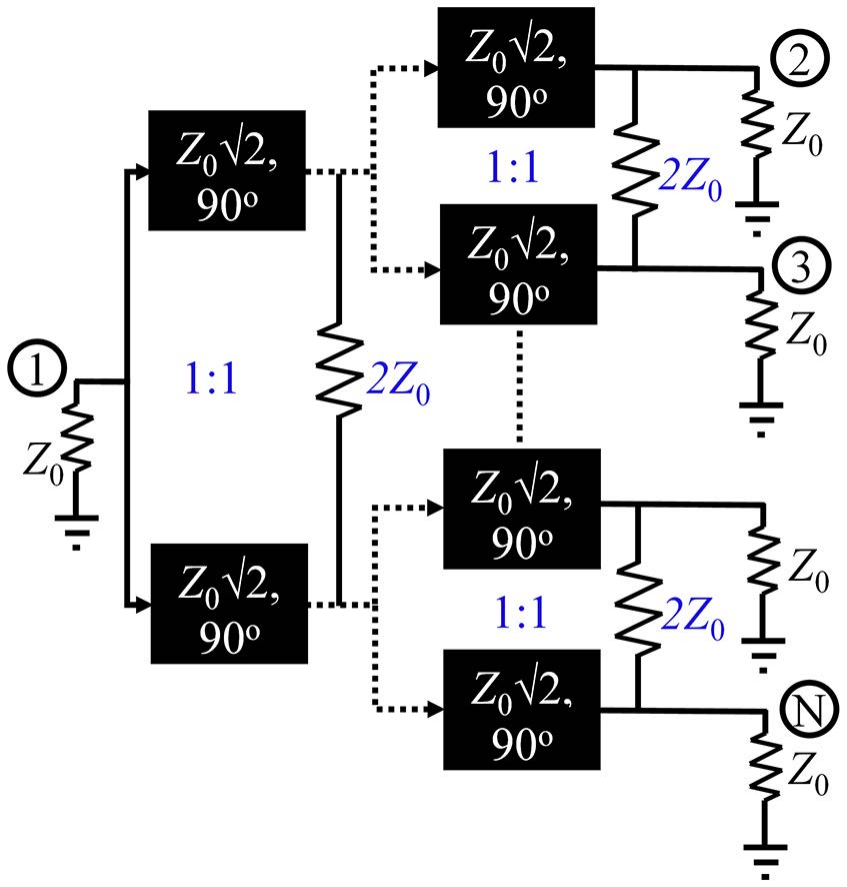
Case B: conventional multistage 1 × *N* equal power WPD topology.

**Fig 10:**
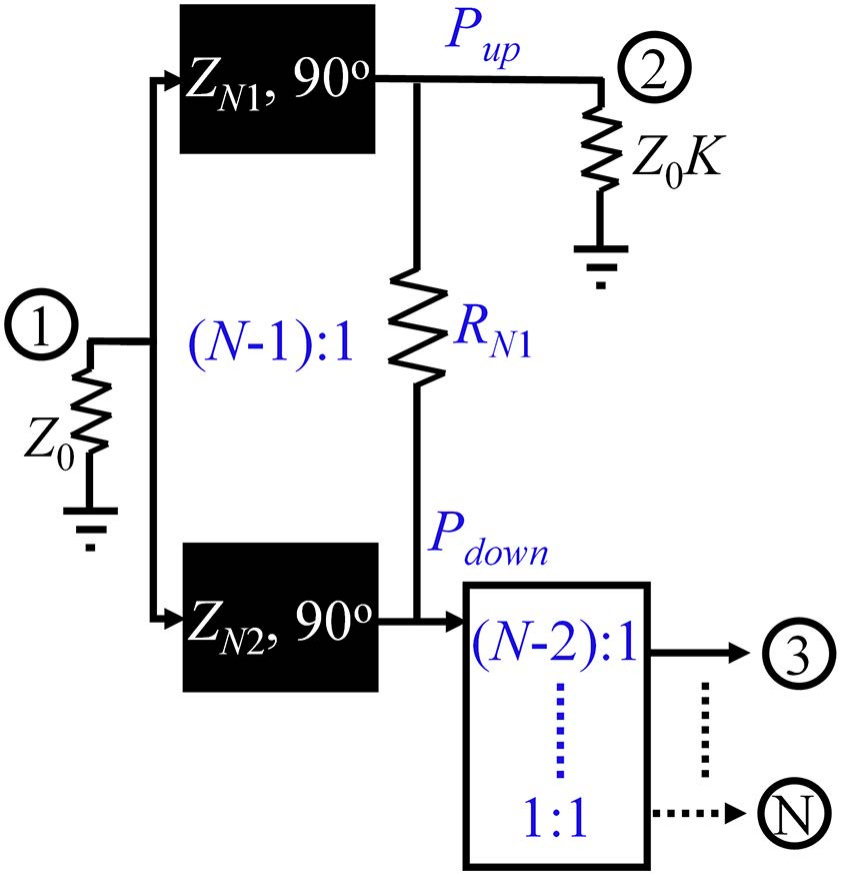
Case C: conventional multistage unequal power 1 × *N* equal power WPD topology.

**Fig 11:**
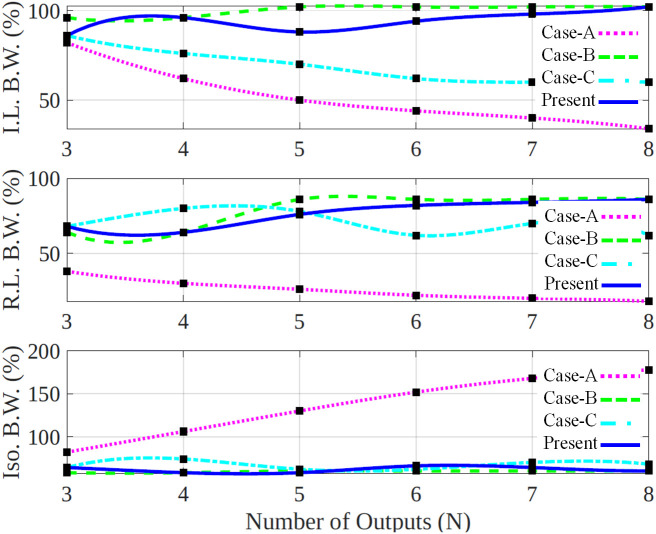
Comparison of I.L., R.L., and Iso. B.W for all four topologies.

**Fig 12:**
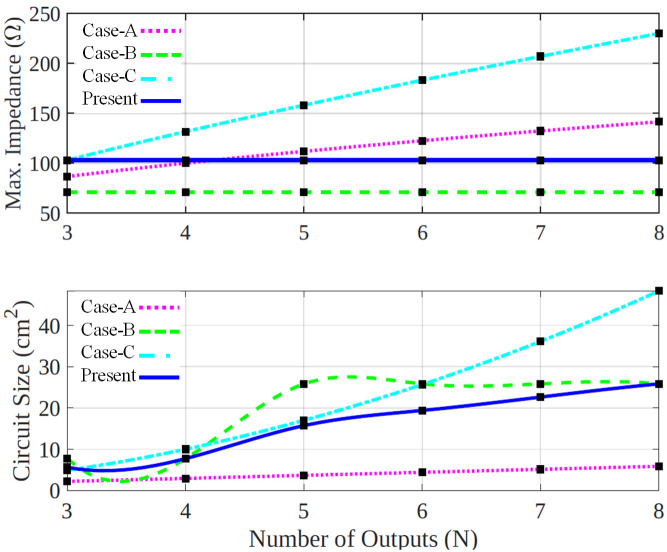
Comparison of max. impedance requirement and circuit size for all four topologies.

Various possible *N*-way equal power WPD topologies as cases A, B and C, are shown in Fig 8,9 and 10, respectively. Comparison graphs of various figure of merits such as insertion loss (I.L.), return loss (R.L.) and isolation (Iso.), bandwidth (B.W.) required for *N* = 3–8, for all topologies, are shown in Fig 11. Similarly, graphs for overall circuit size and maximum line impedance required for *N* = 3–8, for all topologies, are shown in Fig 12. For effective bandwidth calculation, output power magnitude imbalance of ± 0.5 dB and both R.L. and Iso. Magnitudes greater than 15 dB are considered.

The unrealizable impedance requirements of case A beyond 1:6 restrict its use to a maximum of 36 output ports. Additionally, multiple crossovers lead to higher fabrication complexity and larger overall size. Nevertheless, the Iso. Bandwidth improves because of the high isolation resistor, while I.L. and R.L. bandwidths degrade rapidly as outputs increase. Although case B provides comparable bandwidth and impedance requirements, its binary output property results in excessive circuit size with significant unused PCB. For example, a 1 × 9 WPD using this approach contains nearly 50% unused PCB area. Extremely high and low impedance requirements, impractical isolation resistor values, and exponential one-dimensional circuit growth are the key limitations of case C.

The present approach overcomes the limitations of traditional topologies, enabling any number of outputs with easily realizable maximum impedance and practical resistor values without degrading effective bandwidth. Additionally, the entire PCB area is efficiently utilized. For comparison with conventional designs, the limiting case of case A, i.e., *N* = 6, is considered, where impedance requirements for A, B, and C topologies are 122.5 Ω, 70.7 Ω, and 183.2 Ω, and circuit sizes with tapered sections are about 9.5 cm², 25.8 cm², and 36.2 cm², respectively. For the proposed approach, these values are 103 Ω and 19.4 cm², respectively. Thus, miniaturization of 25% and 50% is achieved compared to cases B and C, respectively. Although the circuit size for case A is only 50% of the proposed WPD, its impedance requirement is unrealistically high.

## 4. Design, Fabrication and Measurements

**Fig 13:**
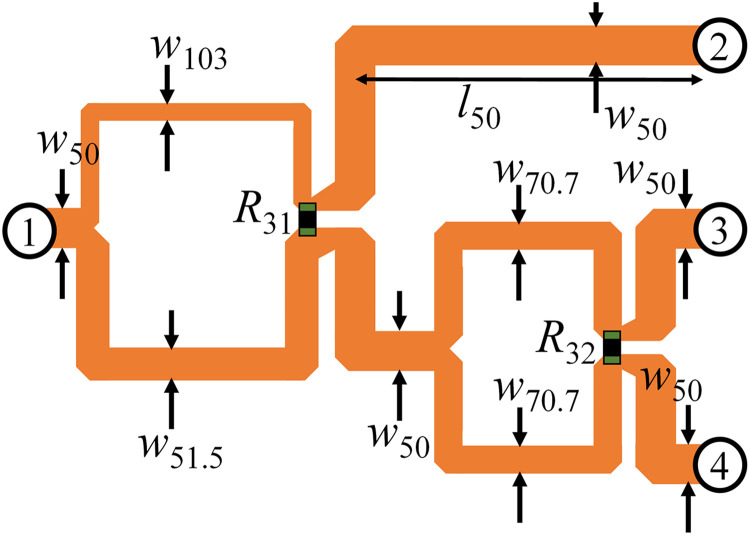
Layout of present 1 × 3 WPD unit (*w*_50_ = 1.14, *w*_103_ = 0.15, *w*_51.5_ = 1.0, *w*_70.7_ = 0.6, *l*_50_ = 14; unit: mm).

**Fig 14:**
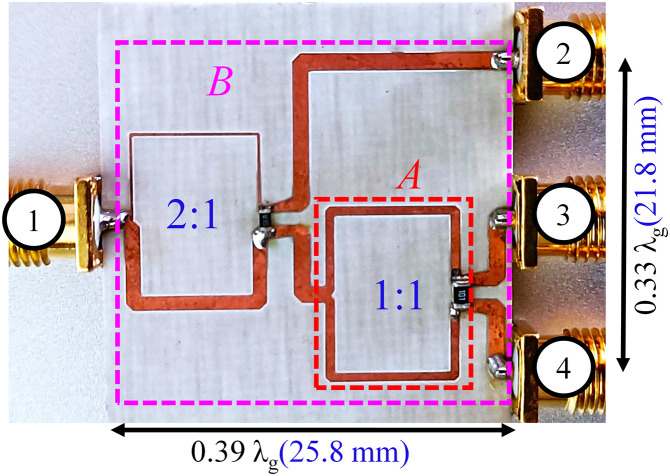
Fabricated prototype of present 1 × 3 WPD unit.

The full wave simulation tool Ansys HFSS is used to obtain the final physical dimensions of power dividers. A 20-mil thick RO4003C substrate with ε_r_ = 3.55, and tanδ = 0.0027 is used for simulations and fabrications. In the present WPD, the first and second stages are designed for 1:2 unequal and 1:1 equal power division, respectively. For verification of basic units, one 1 × 3 WPD is designed with C shape geometry instead of the quarter wavelength arms, along with optimized tapered length to achieve miniaturized WPD. As a result, the overall circuit size of the modified 1 × 3 WPD unit is 5.62 cm^2^, while it is 13.32 cm^2^ using standard quarter wavelength lines. Fig 13 shows the microstrip line layout of the present equal power WPD circuit. Various dimensions are mentioned in the figure caption. The present divider is purposely designed for the phased array application; therefore, at port 2, an additional 50 Ω line of 90° electrical length is added. As a result, the output amplitude at port 2 is reduced. However, equal phase at each output is maintained, which is necessary for phased array applications. A prototype two-stage 1 × 3 Wilkinson power divider in microstrip line technology is fabricated for 2.4 GHz operation, shown in Fig 14.

**Fig 15:**
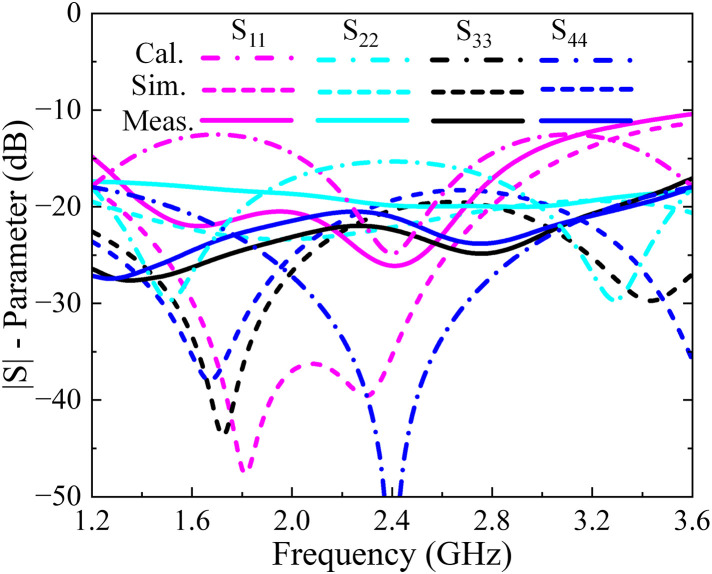
Calculated, simulated and measured reflection coefficient of the present 1 × 3 WPD unit.

**Fig 16:**
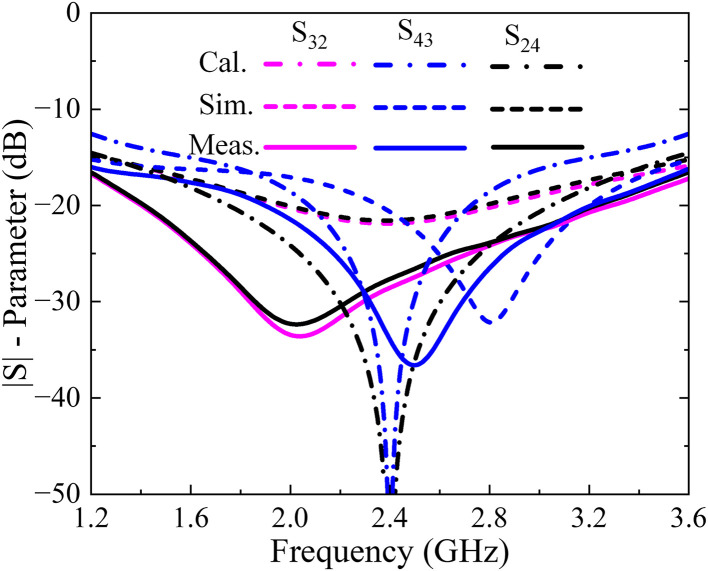
Calculated, simulated and measured isolation of the present 1 × 3 WPD unit.

**Fig 17:**
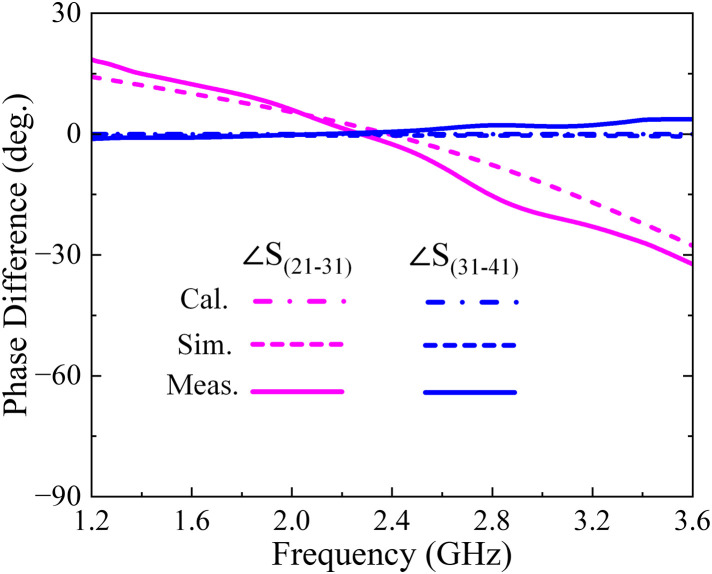
Calculated, simulated and measured phase difference of the present 1 × 3 WPD unit.

**Fig 18:**
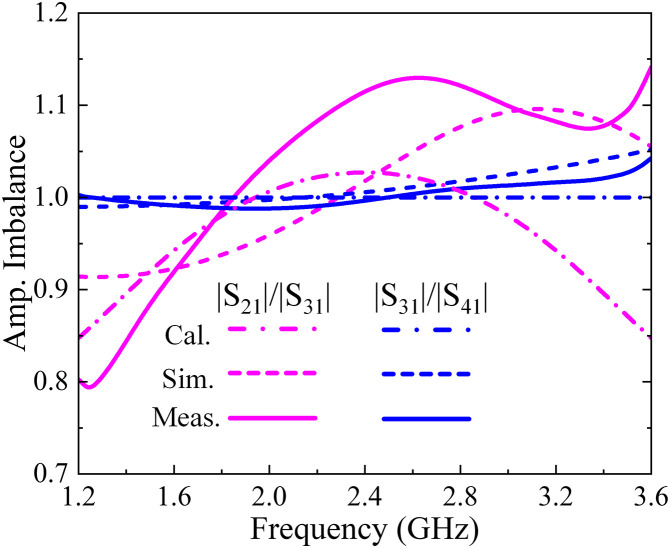
Calculated, simulated and measured amplitude imbalance of the present 1 × 3 WPD unit.

Fig 15 and 16 show calculated, simulated, and measured reflection, transmission coefficient, and isolation plots of WPD, respectively. Measured minimum 15 dB return loss and at least 17 dB isolation between all output ports are achieved over the frequency range of 1.2–3.0 GHz. At the center frequency of 2.4 GHz, these values are a minimum of 20 dB and 22 dB, respectively. Calculated, simulated and measured phase difference and amplitude imbalance between output ports are plotted in Fig 17 and 18. However, a different phase difference slope is seen due to the additional transmission line at port 2; the measured phase difference at the design frequency is within ± 2.5°. Amplitude imbalance is well within ± 0.2 dB over 1.2–3.6 GHz, while it is maximum ± 0.1 dB at 2.4 GHz. However, measured magnitude variations at port 2 deviate from simulated ones; it is well within the acceptable range. The rectangular size occupied by one unit of the present 1 × 3 WPD is 0.13λ^2^_g_ at mid-band frequency.

Performance of the proposed structure is compared to other designs and listed in Table 2. It shows that the present WPD delivers excellent performance with a compact PCB area among all except single-stage WPD configurations [9,12], and [13]. However, single-stage equal power division units in [4,5,8], and [12] offer higher isolation; all other designs offer comparable isolation response. Excellent bandwidth response is shown by the present design except [6], using a smaller-sized circuit. Moreover, the present work demonstrates the least complexity, even for an arbitrary number of outputs across all available designs.

**Table 2:** Comparison with recent wilkinson power dividers works.

Ref./ Year	Technology used	Bandwidth (%)	Size reduction (%)	Insertion Loss (dB)	Isolation (dB)
[3]/ 2007	MSL-EBG	< 50	30.0	3.4	12
[4]/ 2009	MSL	62	37.6	3.3	22
[5]/ 2013	MSL-lumped	27	50.0	3.3	20
[6]/ 2016	MSL-lumped	116	40.0	3.55	30
[7]/ 2021	Slot-line	57.1	20.0	4.25	15
[8]/ 2023	MSL	66.7	45.0	7.3	20
[9]/ 2024	MSL-lumped	78.6	80.0	3.5	16
[10]/ 2016	MSL	14	50.0	4.81, 1.81	15
[11]/ 2017	Artificial TL	30	40.0	7.37, 1.17	15
[12]/ 2023	MSL	73.3	75.0	3.015	20
[13]/ 2024	MSL-lumped	44	84.0	5.12, 2.07	15
**This/ 2026**	**MSL**	**85**	**58.0**	**5.10**	**17**

## 5. Conclusion

A generalized Wilkinson power divider topology for arbitrary *N*-way equal power division is presented here. By combining unequal and equal power division stages, the design achieves compactness, wide bandwidth, and scalability without relying on impractical impedances or unused matched ports. The approach offers significant miniaturization through tapered-line transformers, yielding up to 58% reduction in PCB area compared to conventional multistage topologies, while maintaining an 85% effective bandwidth. A 1 × 3 prototype fabricated at 2.4 GHz confirms the theoretical and simulated results. In the proposed topology, the tapered impedance transformers play a significant role in bandwidth enhancement by providing smoother impedance transitions between interconnected divider sections, thereby reducing reflections over a wider frequency range. The present WPD topology is well-suited for integration in modern phased arrays, wireless LANs, and broadband RF front-end systems. However, the present work focuses on equal power division, the design methodology inherently supports the incorporation of unequal power division stages through appropriate selection and cascading of divider units. This feature enables realization of non-uniform excitation distributions required for sidelobe level reduction techniques such as Taylor, Chebyshev, or other amplitude tapering methods. In principle, the proposed topology is extendable to mm-Wave frequencies due to its transmission line based closed form expression. However, in mm-Wave range, few practical challenges such as pronounced losses, sensitive fabrication tolerance and advanced EM optimization to maintain precise amplitude and phase balance across the output will arise. Despite these challenges, the proposed topology possesses several favourable characteristics for mm-Wave adaptation, including the absence of crossovers, the avoidance of unrealizable high-impedance lines, and a compact, scalable structure. These features can simplify layout complexity in dense multiport beamforming networks.
